# ICTV Virus Taxonomy Profile: *Dicistroviridae*

**DOI:** 10.1099/jgv.0.000756

**Published:** 2017-04-01

**Authors:** S. M Valles, Y Chen, A. E Firth, D. M. A Guérin, Y Hashimoto, S Herrero, J. R de Miranda, E Ryabov, ICTV Report Consortium

**Affiliations:** ^1^​ United States Department of Agriculture, Agricultural Research Service, Gainesville FL 32608, USA; ^2^​ United States Department of Agriculture, Agricultural Research Service, Beltsville MD 20705, USA; ^3^​ Department of Pathology, University of Cambridge, Cambridge CB2 1QP, UK; ^4^​ Department of Biochemistry and Molecular Biology, University of the Basque Country (EHU), Biophysics Institute (CSIC-UPV/EHU), B° Sarriena S/N, 48940 Leioa, Spain; ^5^​ Protein Sciences Corporation, Meriden CT 06450, USA; ^6^​ Department of Genetics, Universitat de València, Burjassot, Spain; ^7^​ Department of Ecology, Swedish University of Agricultural Sciences, Uppsala 750 07, Sweden

**Keywords:** *Dicistroviridae*, ICTV Report, taxonomy

## Abstract

*Dicistroviridae* is a family of small non-enveloped viruses with monopartite, linear, positive-sense RNA genomes of approximately 8–10 kb. Viruses of all classified species infect arthropod hosts, with some having devastating economic consequences, such as acute bee paralysis virus in domesticated honeybees and taura syndrome virus in shrimp farming. Conversely, the host specificity and other desirable traits exhibited by several members of this group make them potential natural enemies for intentional use against arthropod pests, such as triatoma virus against triatomine bugs that vector Chagas disease. This is a summary of the International Committee on Taxonomy of Viruses (ICTV) Report on the taxonomy of the *Dicistroviridae* which is available at www.ictv.global/report/dicistroviridae.

## Virion

**Fig. 1.**
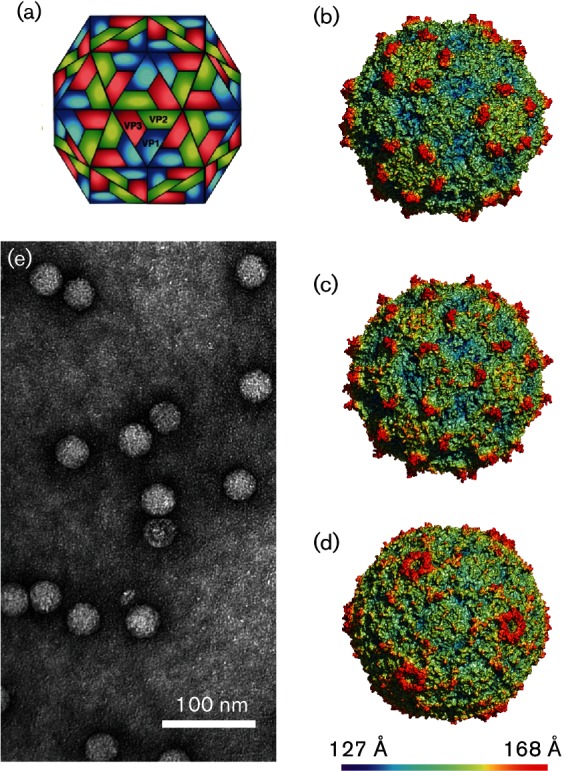
(a) Diagram illustrating the packing of dicistrovirus surface proteins VP1, VP2 and VP3. Renderings (courtesy of A. E. Mechaly) of (b) triatoma virus, (c) Israeli acute bee paralysis virus and (d) cricket paralysis virus (colour scale indicates distance from the particle centre). (e) Negative contrast electron micrograph of purified triatoma virus (courtesy of G. A. Marti).

Virions are roughly spherical, with a particle diameter of approximately 30 nm and no envelope (Table 1, Fig. 1). The virions exhibit icosahedral, pseudo-T=3 symmetry and are composed of 60 protomers, each comprising a single molecule of VP1, VP2 and VP3. A smaller protein, VP4, is also present inside the virion and in contact with the genome [1].

**Table 1.** Characteristics of the family *Dicistroviridae*

Typical member:	cricket paralysis virus (AF218039), species *Cricket paralysis virus*, genus *Cripavirus*
Virion	Non-enveloped, 30 nm-diameter virions
Genome	8–10 kb of positive-sense, non-segmented RNA
Replication	Cytoplasmic within viral replication complexes formed from a variety of host cellular membranes
Translation	Directly from genomic RNA, initiated at IRES elements in the 5′ UTR and IGR
Host range	Arthropoda
Taxonomy	Member of the order *Picornavirales*. Includes the genera *Aparavirus, Cripavirus* and *Triatovirus*, each containing several species

## Genome

**Fig. 2.**
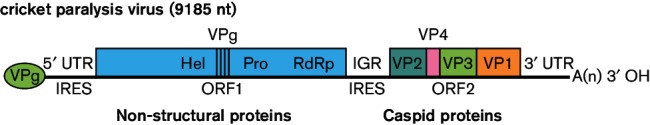
Genome structure of cricket paralysis virus. The RNA genome contains two non-overlapping ORFs separated by an IGR. The 5′ proximal ORF encodes the non-structural proteins: RNA helicase (Hel), cysteine protease (Pro) and RNA-dependent RNA polymerase (RdRp). The structural proteins are encoded by the 3′-proximal ORF.

The RNA genome is monopartite and contains two main non-overlapping ORFs that are flanked by UTRs and separated by an intergenic region (IGR) (Fig. 2). The 5′-proximal and 3′-proximal ORFs encode non-structural and structural protein precursors, respectively. Components of the non-structural polyprotein include an RNA-dependent RNA polymerase, cysteine protease, RNA helicase and one or more copies of a VPg protein. VPg is covalently linked to the 5′ end of the genome.

## Replication

Replication occurs exclusively in the cytoplasm of infected cells. Cap-independent translation proceeds directly from two distinct internal ribosomal entry site (IRES) elements located within the 5′-UTR and the IGR. These IRES elements permit production of non-structural proteins early in the infection process before host translation mechanisms are inhibited, and excess molar quantities of structural proteins when capsid proteins are required later. Unusually, the IGR IRES directs translation initiation at a 3′-adjacent non-AUG codon and in the absence of all canonical initiation factors. The conserved three-dimensional structure is crucial to the IGR IRES function [2]. Pseudo-knot and stem-loop structures in the IGR IRES are highly conserved across all members of the family and facilitate interactions with the ribosome [3]. The 5′-UTR IRES is not obviously conserved in sequence or structure across the group. Translation activity of the IGR IRES is comparatively greater than that of the 5′-UTR IRES at late time points [4].

## Taxonomy

The family *Dicistroviridae* is comprised of three genera: *Aparavirus*, *Cripavirus* and *Triatovirus*. Demarcation of the genera is based on phylogenetic divergence and unique characteristics exhibited by the internal ribosomal entry site [2]. Dicistrovirus infections vary considerably in virulence and pathogenicity; the severity of disease ranges from inapparent to lethal. Transmission via ingestion and the alimentary canal feature prominently in dicistrovirus infection acquisition and transmission. Most of the dicistroviruses exhibit a tissue tropism toward some part of the alimentary canal, often replicating in epithelial cells of the gut and subsequently shedding virus particles into the gut lumen where virus accumulates in the faeces and serves as inoculum [5]. Nervous tissue, fat body, epidermal cells and gonads may also support replication of dicistroviruses.

## Resources

Full ICTV Online (10th) Report: www.ictv.global/report/dicistroviridae.
